# Integrated Taxonomy and DNA Barcoding of Alpine Midges (Diptera: Chironomidae)

**DOI:** 10.1371/journal.pone.0149673

**Published:** 2016-03-03

**Authors:** Matteo Montagna, Valeria Mereghetti, Valeria Lencioni, Bruno Rossaro

**Affiliations:** 1 Dipartimento di Scienze Agrarie e Ambientali—Università degli Studi di Milano, Via Celoria 2, I-20133, Milano, Italy; 2 MUSE—Museo delle Scienze, Corso del Lavoro e della Scienza 3, I-38122, Trento, Italy; 3 Dipartimento di Scienze per gli Alimenti, la Nutrizione e l’Ambiente—Università degli Studi di Milano, Via Celoria 2, I-20133, Milano, Italy; Nanjing Agricultural University, CHINA

## Abstract

Rapid and efficient DNA-based tools are recommended for the evaluation of the insect biodiversity of high-altitude streams. In the present study, focused principally on larvae of the genus *Diamesa* Meigen 1835 (Diptera: Chironomidae), the congruence between morphological/molecular delimitation of species as well as performances in taxonomic assignments were evaluated. A fragment of the mitochondrial *cox1* gene was obtained from 112 larvae, pupae and adults (Diamesinae, Orthocladiinae and Tanypodinae) that were collected in different mountain regions of the Alps and Apennines. On the basis of morphological characters 102 specimens were attributed to 16 species, and the remaining ten specimens were identified to the genus level. Molecular species delimitation was performed using: *i*) distance-based Automatic Barcode Gap Discovery (ABGD), with no *a priori* assumptions on species identification; and *ii*) coalescent tree-based approaches as the Generalized Mixed Yule Coalescent model, its Bayesian implementation and Bayesian Poisson Tree Processes. The ABGD analysis, estimating an optimal intra/interspecific nucleotide distance threshold of 0.7%-1.4%, identified 23 putative species; the tree-based approaches, identified between 25–26 entities, provided nearly identical results. All species belonging to *zernyi*, *steinboecki*, *latitarsis*, *bertrami*, *dampfi* and *incallida* groups, as well as outgroup species, are recovered as separate entities, perfectly matching the identified morphospecies. In contrast, within the *cinerella* group, cases of discrepancy arose: *i*) the two morphologically separate species *D*. *cinerella* and *D*. *tonsa* are neither monophyletic nor diagnosable exhibiting low values of between-taxa nucleotide mean divergence (0.94%); *ii*) few cases of larvae morphological misidentification were observed. Head capsule color is confirmed to be a valid character able to discriminate larvae of *D*. *zernyi*, *D*. *tonsa* and *D*. *cinerella*, but it is here better defined as a color gradient between the setae submenti and genal setae. DNA barcodes performances were high: average accuracy was ~89% and precision of ~99%. On the basis of the present data, we can thus conclude that molecular identification represents a promising tool that could be effectively adopted in evaluating biodiversity of high-altitude streams.

## Introduction

Recent climatic warming has had a strong impact on habitats and species occurring at high elevation [1]. Amongst other effects, extensive environmental change occurs during glacial retreat, which affects hydrological and thermal regimes of glacier-fed streams [2]. Stream biodiversity is expected to dramatically change in relation to retreating glaciers, favoring an upstream shift of lowland species associated with local extinction of kryal species [3–7]. Unfortunately, a monitoring method and dedicated biotic indices, able to evaluate the biotic components of high-altitude habitats, have not yet been developed. There are several reasons for this, including and most importantly difficulties in identifying larvae belonging to the genus *Diamesa* Meigen 1835 (Diptera; Chironomidae), which dominate species richness and abundance in glacial streams and cold spring habitats [7–9]. Identification of midges based on morphology can be accurately achieved for adult males [10–11] or to a lesser extent for pupal exuviae [10], as clear discriminating features are visible for these stages. In addition, at present, despite updated keys identifying *Diamesa* larvae being available [12], the implementation of innovative tools, able to accurately identify larvae of the genus *Diamesa* by integrating different sources of information (e.g., molecular and morphological diagnostic characters), are required. Such approaches will promote the exploitation of ecological information provided by the presence/absence of these species in the habitats under study [3].

The West Palaearctic species belonging to the genus *Diamesa* have been separated into nine different groups [13] according to adult male and pupal morphology. A combination of qualitative and quantitative characters observable in larvae (head capsule color, mouth-parts and posterior body appendages) only partially confirm this separation. This is because *D*. *aberrata* Lundbeck, 1898 and *D*. *incallida* (Walker 1856), included by Serra-Tosio in the *aberrata* group, have very different larvae, suggesting the separation into two distinct groups, while *D*. *bertrami*, Edwards, 1935, included by Serra-Tosio in the *zernyi–insignipes–cinerella* groups, has a larva very similar to the larvae of the *latitarsis* group [12]. Within each group, determination to species level is generally hampered by the lack of diagnostic characters or by the degradation of valid taxonomic characters, such as mental and mandibular teeth, in field-collected samples [12]. Moreover, quantitative characters should be used with caution due to the intraspecific variability (even within the fourth larval instar) present in different populations adapted to different environmental conditions [14].

Larvae belonging to *zernyi–insignipes–cinerella* groups [13] share the presence of a very reduced procercus bearing four anal setae of moderate length (~200–300 μm) and short posterior pseudopods [12, 15]. At present, species belonging to these groups are separated from each other only according to head capsule color, from yellow (*D*. *insignipes* Kieffer in Kieffer and Thienemann 1908 and *D*. *cinerella* Meigen in Gisti 1835) to dark brown (*D*. *zernyi* Edwards 1933 and *D*. *vaillanti* Serra-Tosio 1972). *D*. *tonsa* (Haliday in Walker 1856) represents an intermediate case, possessing a yellow head capsule with variably extended brown areas [12, 15].

The larvae belonging to *steinboecki*, *latitarsis*, *bertrami* and *aberrata* groups differ to those of the *zernyi–insignipes*–*cinerella* group as they possess very elongated posterior pseudopods, reduced anal setae (<120 μm), and are characterized by the absence of procerci and a dark head capsule. In addition, identification of species belonging to these groups is extremely difficult, despite recent proposal of valid diagnostic traits such as the number, length and diameter of anal setae and the shape of the mentum [12]. Only the *dampfi* and *incallida* groups are easily separated from other species at the larval stage: the former due to the presence of well developed procerci bearing six anal setae, the latter due to the characteristic annulation of the third antennal segment.

The present study is designed to test the congruence between species identifications on the basis of morphological diagnostic characters (i.e., morphospecies), such as head capsule color and anal setae length [12, 15, 16], and putative molecular species (operational taxonomic units, evolutionary species and phylospecies) delimitated using different approaches on the basis of a single-gene marker (i.e., the mitochondrial cytochrome oxidase subunit I–*cox1*). In addition, the effectiveness of DNA barcoding for species-level identification is tested. In recent years, molecular-based approaches have been successfully adopted to delimit midge species [17, 18] and facilitate species identification (e.g., DNA barcoding studies). Contrasting results have been achieved by DNA barcoding, including cases in which its utility has been demonstrated [17–24] and others in which the adopted approach failed to identify the species [25, 26].

## Materials and Methods

### Ethics Statement

No species of Diptera Chironomidae are listed in any national or regional law as protected or endangered. All the specimens were collected in state-owned properties. The collection of these invertebrates is not subjected to restriction by Italian law and does not require permission; permission to collect biological specimens in protected areas was provided by the competent authorities (prot. N. 2342/V/8/2-2014; prot. N. 2598/10.10–2015).

### Sampling, Specimen Manipulation and Morphological Identification

**Fig 1:**
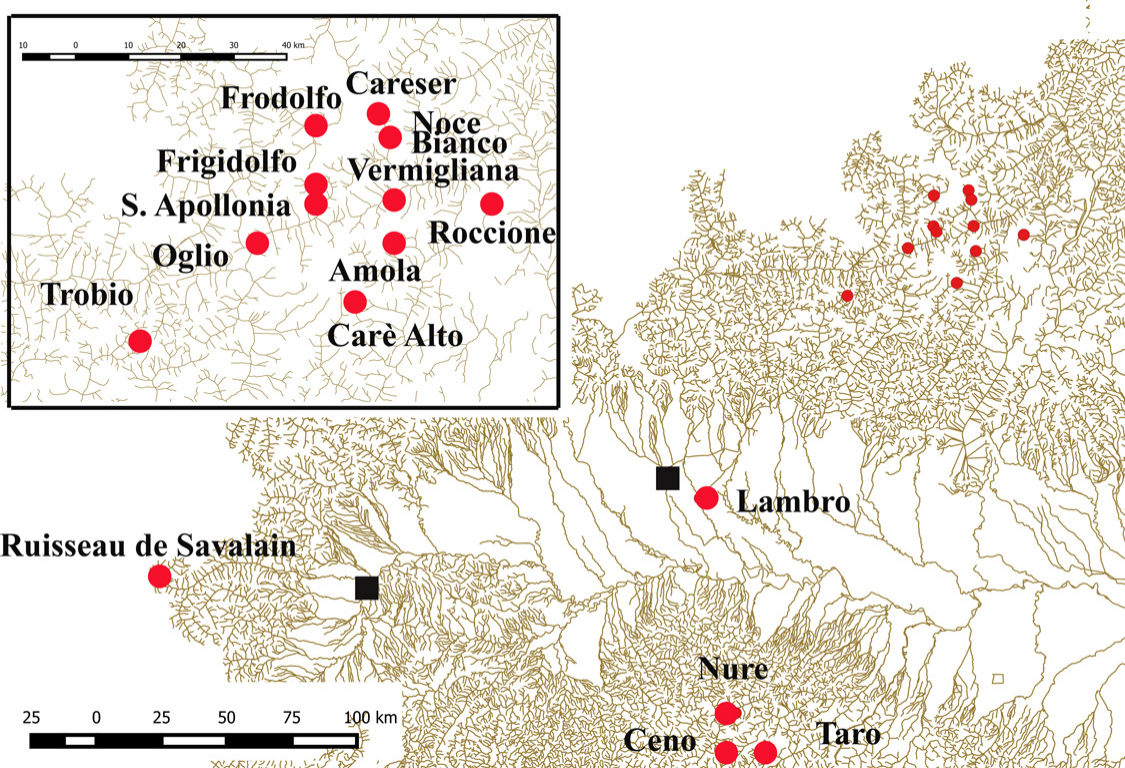
Geographical location of collecting sites in Northern Italy. Localities in which samples were collected are denoted by red dots while black squares indicate the cities of Turin (to the west) and of Milan (towards the center of the map). The inset shows a magnification of the collecting localities within the Rhaetian Alps.

Chironomid samples (larvae, pupae and adults) were collected by using drift net, Surber net and malaise traps during several field trips between 2013 and 2015 in nine localities within the Alps and Apennines (Table 1; Fig 1). The collected specimens were directly placed in absolute ethanol and sorted to the genus level in the laboratory by stereomicroscopy (Leica DM LS B2 and Leica MS 5). DNA was extracted from the body of full-grown larvae (4^th^ instar) after the removal of the head capsule and the caudal part, while DNA was extracted from pupae and adults preserving the whole morphology. The removed larval parts were mounted on a microscope slide in Canada Balsam, after dehydration with acetic acid and clarification with phenol-xylene 3:1 [27, 28], then identified to the species level [12] on the basis of morphological features including all semaphoronts (adults, pupae and larvae; morphological species concept), whenever possible. Pupae and adult males were mounted as usual, and identified using available identification keys [10, 29]. Species ecology and distribution, as well as association of larvae with pupae and adults collected in the same locality were also considered as additional information to identify the specimens, e.g., adult males of *D*. *insignipes* have never been collected within the Alps [3], and so larvae with a yellow head were not assigned to this species. Measures were acquired by using optical microscopy at different magnifications (× 40 – × 1000), including photography using a digital camera (Leica DFC320).

**Table 1:** Analyzed species of chironomids.

Collecting site	Source	m a.s.l.	Lat N	Long E	Species	Sample ID
I-TN-Vermiglio	Vermigliana^t^	1350	46°16'28"	10°38'59"	*D*. *tonsa*	37^¶^
	Vermigliana^t^	1350	46°16'28"	10°38'59"	*D*. *zernyi*	14^●^
	Vermigliana^t^	1350	46°16'28"	10°38'59"	*Diamesa* sp. 3	13, 36^○^
I-TN-Vermiglio	Vermigliana^t^	1210	46°17'8"	10°40'21"	*D*. *tonsa*	90^¶^
I-TN-Tuenno, Tovel Lake	Roccione^s^	1220	46°15'39"	10°57'28"	*D*. *tonsa*	16^¶^-18^¶^
	Roccione^s^	1220	46°15'39"	10°57'28"	*D*. *zernyi*	40^●^
	Roccione^s^	1220	46°15'39"	10°57'28"	*Pseudokiefferiella parva*	15
I-TN-Amola glacier	Amola^t^	2420	46°12'47"	10°42'24"	*D*. *bertrami*	53–55, 99
	Amola^t^	2420	46°12'47"	10°42'24"	*D*. *cinerella*	96n
	Amola^t^	2380	46°12'37"	10°42'35"	*D*. *cinerella*	110e
	Amola^t^	2540	46°13'01"	10°41'41"	*D*. *goetghebueri*	20, 21, 34
	Amola^t^	2420	46°12'47"	10°42'24"	*D*. *goetghebueri*	22–26, 29, 61, 62
	Amola^t^	2420	46°12'47"	10°42'24"	*D*. *incallida*	38
	Amola^t^	2540	46°13'01"	10°41'41"	*D*. *tonsa*	59^¶^, 60^○^
	Amola^t^	2540	46°13'01"	10°41'41"	*D*. gr. *tonsa* 3^rd^ in.	47^¶^-49^¶^
	Amola^t^	2540	46°13'01"	10°41'41"	*D*. *steinboecki*	19, 30–33, 46
	Amola^t^	2420	46°12'47"	10°42'24"	*D*. *steinboecki*	28, 56–58, 95♂
	Amola^t^	2420	46°12'47"	10°42'24"	*D*. *zernyi*	97rn, 98^●^
	Amola^t^	2420	46°12'47"	10°42'24"	*Diamesa* sp. 1	98^●^
	Amola^t^	2450	46°12'51"	10°42'89"	*D*.*iamesa* sp. 2	113
I-TN-Carè Alto glacier	Conca^t^	2510	46°06'05"	10°37'01"	*D*. *dampfi*	51, 52
	Conca^t^	2510	46°06'05"	10°37'01"	*D*. *latitarsis*	50
I-TN-de la Mare glacier	Noce bianco^t^	1740	46°24'23"	10°41'45"	*D*. *zernyi*	114n
I-TN-Careser glacier	Careser^t^	2650	46°25'52"	10°42'25"	*D*. *goetghebueri*	93, 94
I-TN-Careser glacier	Careser^t^	2650	46°25'52"	10°42'25"	*D*. *cinerella*	106er
I-PR-Compiano	Taro^r^	510	44°29'39"	9°39'28"	*D*. *tonsa*	63n
I-PR-Piane di Carniglia	Taro^r^	500	44°29'7"	9°37'4"	*O*. (*O*.) *glabripennis*	64
	Taro^r^	519	44°29'7"	9°37'4"	*S*. *spinifera*	65–67
I-PR-Anzola	Ceno^t^	780	44°31'29"	9°33'22"	*O*. (*O*.) *glabripennis*	74
	Ceno^t^	780	44°31'29"	9°33'22"	*O*. (*E*.) *rivulorum*	70, 71
	Ceno^t^	780	44°31'29"	9°33'22"	*S*. *spinifera*	68, 69, 73
I-BS-Vezza d’Oglio	Oglio^r^	1070	46°14'26"	10°23'50"	*D*. *modesta*	6
	Oglio^r^	1070	46°14'26"	10°23'50"	*D*. *tonsa*	3^¶^, 7^¶^-9, 11^¶^, 39^¶^
	Oglio^r^	1070	46°14'26"	10°23'50"	*Macropelopia* sp.	2
	Oglio^r^	1070	46°14'26"	10°23'50"	*O*. (*E*.) spp.	5, 12
	Oglio^r^	1070	46°14'26"	10°23'50"	*Pseudodiamensa* sp.	4
I-BS-Ponte di Legno	spring	1600	46°17'60"	10°30'16"	*Macropelopia* sp.	1
	stream	1600	46°17'60"	10°30'16"	*Pseudodiamensa* sp.	45
	spring	1600	46°17'60"	10°30'16"	*D*. *incallida*	43
	spring/stream	1600	46°17'60"	10°30'16"	*D*. *tonsa*	42^¶^, 44^●^
	spring	1600	46°17'60"	10°30'16"	*O*. (*M*.) *frigidus*	41
I-BS-Ponte di Legno	Frigidolfo^t^	1600	46°17'60"	10°30'16"	*D*. *cinerella*	109^○^
	Frigidolfo^t^	1600	46°17'60"	10°30'16"	*D*. *dampfi*	102, 105
	Frigidolfo^t^	1600	46°17'60"	10°30'16"	*D*. *tonsa*	101^¶^, 103^¶^, 108^¶^
	Frigidolfo^t^	1600	46°17'60"	10°30'16"	*D*. *zernyi*	100^●^, 104^●^, 107^●^
I-SO-Forni glacier	Frodolfo^t^	1770	46°24'30"	10°30'27"	*D*. *cinerella*	84^○^
	Frodolfo^t^	1770	46°24'30"	10°30'27"	*D*. *dampfi*	78, 80
	Frodolfo^t^	1770	46°24'30"	10°30'27"	*D*. *latitarsis*	89
	Frodolfo^t^	1770	46°24'30"	10°30'27"	*D*. *modesta*	75, 77, 79, 85–88
	Frodolfo^t^	1770	46°24'30"	10°30'27"	*D*. *tonsa*	76^¶^, 82^¶^, 83^¶^
	Frodolfo^t^	1770	46°24'30"	10°30'27"	*D*. *zernyi*	81^●^
I-BG-Trobio glacier	Trobio^t^	1950	46°04'03"	10°03'94"	*D*. *vaillanti*	115ll
	Trobio^t^	2360	46°03'43"	10°04'43"	*D*. *goetghebueri*	91, 92
I-PC-Ferriere	Nure^t^	650	44°38'08"	9°29'43"E	*S*. *spinifera*	72
I-TO-Moncenisio Pass	Ruisseau de Savalain^t^	2010	45°14'06"	6°54'09"	*D*. *bertrami*	117
	Ruisseau de Savalain^t^	2010	45°14'06"	6°54'09"	*D*. *zernyi*	116^●^

Note: toponym, altitude, geographical coordinates, water type, specimen identification and identifiers (MR as acronym of Montagna-Rossaro collection is omitted) are reported.

^g^ glacier

^l^ lake

^r^ river

^s^ spring

^t^ torrent

♂ male

P pupa

overall color of head capsule yellow (^○^)

yellow with extended brown areas (^¶^) and

dark brown (^●^).

### DNA Extraction, PCR Amplification and Sequencing

DNA was extracted using DNeasy Blood and Tissue Kit (Qiagen, Heidelberg) following the manufacturer’s instructions. A fragment of 658 bp of the mitochondrial *cox1* gene was amplified by PCR using universal primers for metazoa LCO1490/HCO2198 [30]. The concentration of reagents used for *cox1* amplification and thermal profile followed [31]. Successful amplification was determined by gel electrophoresis and PCR products were bidirectionally sequenced by ABI technology (Applied Biosystems, Foster City, CA, USA). The obtained electropherograms were manually edited and assembled into a consensus sequence using Geneious Pro 5.3 (Biomatters Ltd., Auckland, New Zealand); consensus sequences were deposited in ENA archive (accession numbers: LN897576-LN897687).

### Bioinformatic and Species Delimitation Analyses

The obtained *cox1* gene sequences were checked and aligned at the amino acid level using MUSCLE [32] and then back translated to the nucleotide sequence. The previously obtained alignment was used as input for species delimitation analyses. Independent methods requiring no *a priori* information on the existing morphospecies were adopted: *i*) the automatic barcode gap discovery tool (ABGD; [33]), which attempts to delimit species (here equivalent to operational taxonomic units) by estimating the optimal distance threshold (OT) for the given set of data; *ii*) coalescent tree-based methods as the generalized mixed Yule-coalescent model (GMYC; [34, 35]) associated with its Bayesian implementation (bGMYC) [36] and the Poisson tree process model (PTP; [37]) in order to identify phylospecies and evolutionary species. Molecular approaches delimiting evolutionary units have been successfully adopted in several case studies in insects [38–40]. ABGD analyses were performed using the web-based interface (http://wwwabi.snv.jussieu.fr/public/abgd) with the Kimura-2-parameter model (K2P; [41]) as the model of nucleotide evolution. Prior maximum divergence of intraspecific diversity was settled from the value corresponding to a single nucleotide difference (i.e., 0.00153) to 0.1, relative gap width of 0.5. The remaining parameters were left with default settings. Despite the extensive use of K2P nucleotide distance in the scientific literature, this measure be inadequate to properly delimit species [42–43]. In order to avoid such problems a further ABGD analysis was performed, adopting uncorrected nucleotide distance and the results were compared with those of previous analyses.

The single threshold GMYC method was implemented in the R package "splits" (SPecies LImits by Threshold Statistics) while the bGMYC method was performed in the R package "bGMYC". Bayesian inference analysis was performed by MrBayes 3.2 [44] in order to obtain the phylogram used, after conversion in ultrametric (see below for the adopted procedure), as input for GMYC and bGMYC analyses. Nucleotide substitution models were estimated using jModelTest 2 [45] and the model best-fitting the sequence was selected as General Time Reversible (GTR; [46]) with gamma distribution and proportion of invariable sites according to Bayesian Information Criterion. Two independent runs were performed using the following parameters: length of the Markov chain settled to 1*10^8^ generations; trees and parameters sampled every 1000 generations; models of nucleotide evolution as obtained by model selection. The convergence of the two runs was checked using Tracer [47] and the burn-in fraction estimated accordingly. The Bayesian majority-rule consensus tree was converted to ultrametric in r8s 1.7 [48] using penalized likelihood with a smoothing parameter of 0.1, selected after cross-validation (as described in [38, 49]). The coalescent tree-based PTP method was performed using the web interface available at http://species.h-its.org/ptp/ with the following parameters: the Bayesian majority-rule consensus non-ultrametric tree as input, 5*10^5^ MCMC generations, thinning every 100 generations, burning fraction = 0.20.

Maximum likelihood tree was inferred, adopting previous model parameters and approximate likelihood ratio test as node support (aLRT; [50]), by using PhyML [51].

### DNA Barcoding and Nucleotide Distance Matrix

In its original meaning, DNA barcoding is designed to identify organisms on the basis of a DNA sequence adopting a fixed threshold of nucleotide distance [52]. In order to increase the success of specimen identification, the optimal intra-interspecific nucleotide distance threshold (OT; [53, 54]) estimated from the analyzed dataset of sequences was adopted instead of a fixed, *a priori* defined, threshold. OT corresponds to the values of nucleotide distances at which the sum of false positive (FP; type I errors) and false negative (FN; type II errors) identifications reached minimum values.

All DNA barcoding analyses, including OT optimization, were performed on different *cox1* sequence datasets (hereafter reported as *ds* plus a number from one to six on the basis of their features) using functions implemented in the R package "spider" [55]. For each dataset, a K2P [41] distance matrix was calculated. The accuracy and precision of DNA barcoding was calculated on the basis of the obtained data as defined by [54].

Pairwise nucleotide mean distance, box plots and heat map were estimated using the R package *vegan* [56], K2P [41] was adopted as the model of nucleotide substitutions.

### New Diagnostic Character and Image Analysis

A new diagnostic character, represented by the brown-yellow color gradient in the area joining setae submenti and genal setae [57], has been considered as operative in identifying larvae of *D*. *zernyi*, *D*. *tonsa* and *D*. *cinerella*. The ventral and dorsal part of the head capsule were separated with fine tungsten needles and mounted so that the area between setae submenti and genal setae was easily visible. The RGB color profile of the segment joining setae submenti and genal setae was analyzed using the functions imread.m, imshow.m and improfile.m from the Image Analysis toolbox of Matlab® vers. R2015a.

## Results and Discussion

### Morphological Identification of Analyzed Specimens

**Fig 2:**
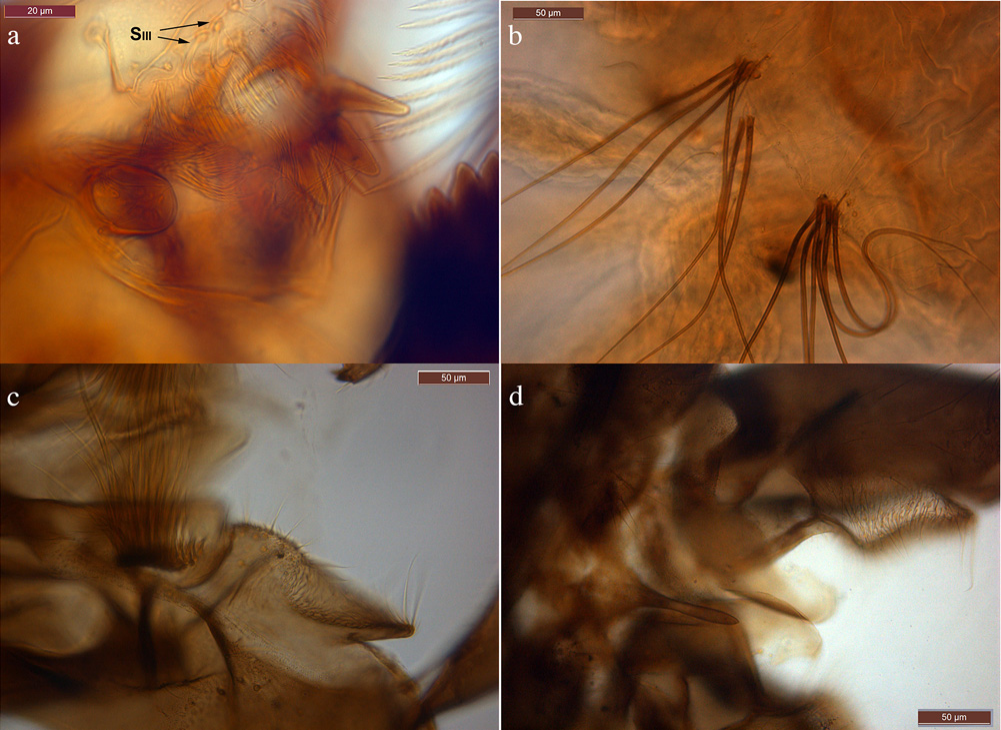
Micrographs of contrasting morphological characters harbored by the three discussed specimens. The upper micrographs report the morphological characters of MR-13: labrum with bifid S_III_ setae (top left) and procercus with six anal setae (top right). Below are micrographs reporting details of the hypopygium, respectively of MR-115 (bottom left) and of MR-113 (bottom right) specimens.

The DNA was extracted from a total of 112 specimens (subfamily Diamesinae, and few Orthocladiinae and Tanypodinae as outgroups) collected in 16 localities in the Alps and Apennines (Fig 1). Morphological identification, geographical coordinates and altitude of collecting localities, developmental stages and the overall head capsule color (the last feature only for larvae belonging to *zernyi* and *cinerella* groups) are reported in Table 1. The 112 specimens analyzed belong to six genera of midges: *Macropelopia* Thienemann 1916, *Diamesa* Meigen 1835, *Pseudodiamesa* Goetghebuer 1939, *Pseudokiefferriella* Zavrel 1941; *Sympotthastia* Pagast 1947 and *Orthocladius* van der Wulp 1874. The species belonging to genera other than *Diamesa* were included in the analyses as outgroups. Among the 93 specimens ascribed to the genus *Diamesa*, 89 are morphologically attributed to the following eleven species: *D*. *bertrami*, *D*. *cinerella*, *D*. *dampfi* (Kieffer 1924), *D*. *goetghebueri* Pagast 1947, *D*. *incallida*, *D*. *latitarsis* (Goetghebuer 1921), *D*. *modesta* Serra-Tosio 1968, *D*. *steinboecki* (Goetghebuer 1921), *D*. *tonsa*, *D*. *vaillanti* Serra-Tosio 1972 and *D*. *zernyi*. In the case of *D*. *tonsa*, *D*. *cinerella*, *D*. *zernyi*, *D*. *goetghebueri*, *D*. *bertrami*, *D*. *steinboecki* and *D*. *vaillanti* adult males and pharate pupae, of unequivocal attribution, are present. Larvae belonging to the *latitarsis* group were identified from adult specimens collected from the same localities. Regarding the remaining four specimens: MR-36 is a larva with a yellow head capsule, which could be identified as *D*. *cinerella*; MR-98 showed a black head and could be identified as *D*. *zernyi*; while MR-13 and MR-113 possess intriguing features. MR-13 is a larva collected from the River Vermigliana (Baita Velon, Trento; Table 1) exhibiting a yellow head capsule but harboring contrasting characters that hampered its identification. MR-13 exhibits six setae on each procercus and bifid S_III_ setae on the labrum (Fig 2), the former feature suggests that this specimen should belong to the *dampfi* group while the latter suggests its ascription to the *zernyi-cinerella* groups. MR-113 is an adult male collected at the Amola glacier that, on the basis of morphological characters, resembles *D*. *nowickiana* Kownacki & Kownacka 1975 (Fig 2).

### Species Delimitation Analyses

A fragment of 658 bp of the mitochondrial *cox1* gene was obtained from 112 specimens (16 identified morphospecies and 10 specimens identified at genus level); no indels were observed.

The aligned *cox1* gene sequences were subjected to ABGD analysis designed to delimit species estimating the OT from the data. The frequency distributions of pairwise K2P distance highlighted the existence of a clear gap in pairwise comparisons (Fig A, B in S1 File). The perfect match between the initial and the recursive partitions occurred at nucleotide divergence values ranging from 0.7% to 1.4% and twenty-three groups (or putative molecular species) were identified (Fig C in S1 File). ABGD analysis implementing the observed nucleotide distance lead to comparable results and 26 groups were identified at the match between initial and recursive partitions. These results showed a high level of congruence between groups identified on the basis of *cox1* gene sequences and the identified morphospecies. Specifically, all species belonging to *D*. *steinboecki*, *latitarsis*, *bertrami*, *dampfi* and *incallida* as well as all outgroup species are recovered as members of separate entities by ABGD analysis performed with K2P distance, perfectly matching morphospecies. Whereas, within the *zernyi–cinerella* groups, all specimens morphologically identified as *D*. *zernyi*, *D*. *tonsa* and *D*. *cinerella* where grouped into two clusters: *i*) a group composed by specimens identified as *D*. *tonsa* and *D*. *cinerella* (including adult male of both species), five unidentified larvae at the 3^rd^ developmental stage, a larva identified as *D*. *zernyi* according to head capsule color (MR-9) and a male pupa identified as *D*. *vaillanti* (MR-115; Fig 2); *ii*) a group composed of specimens of *D*. *zernyi* (with an adult male), all larvae initially identified as *D*. *zernyi* on the basis of the overall color of the head capsule. Thus, on the basis of the adopted distance-based approach, *D*. *tonsa–D*. *cinerella–D*. *vaillanti* (only one) specimens of certain morphological identification (adult males and larvae of clear attribution) belong to the same unit. Interestingly, the specimen MR-13 showed contrasting characters (MR-13; Fig 2) and, being collected from the River Vermigliana clustered with MR-36 from the same locality, in a single, separate group. ABGD analysis, performed using observed nucleotide distance, identified the specimens *D*. *zernyi* MR-40, *D*. *steinboecki* MR-32 and *D*. *tonsa* MR-108 as entities separated from groups harboring conspecific specimens. Putative molecular species recovered by ABGD analysis adopting the K2P model of evolution are more congruent with morphology with respect to those achieved by the same approach using observed nucleotide distance.

**Fig 3:**
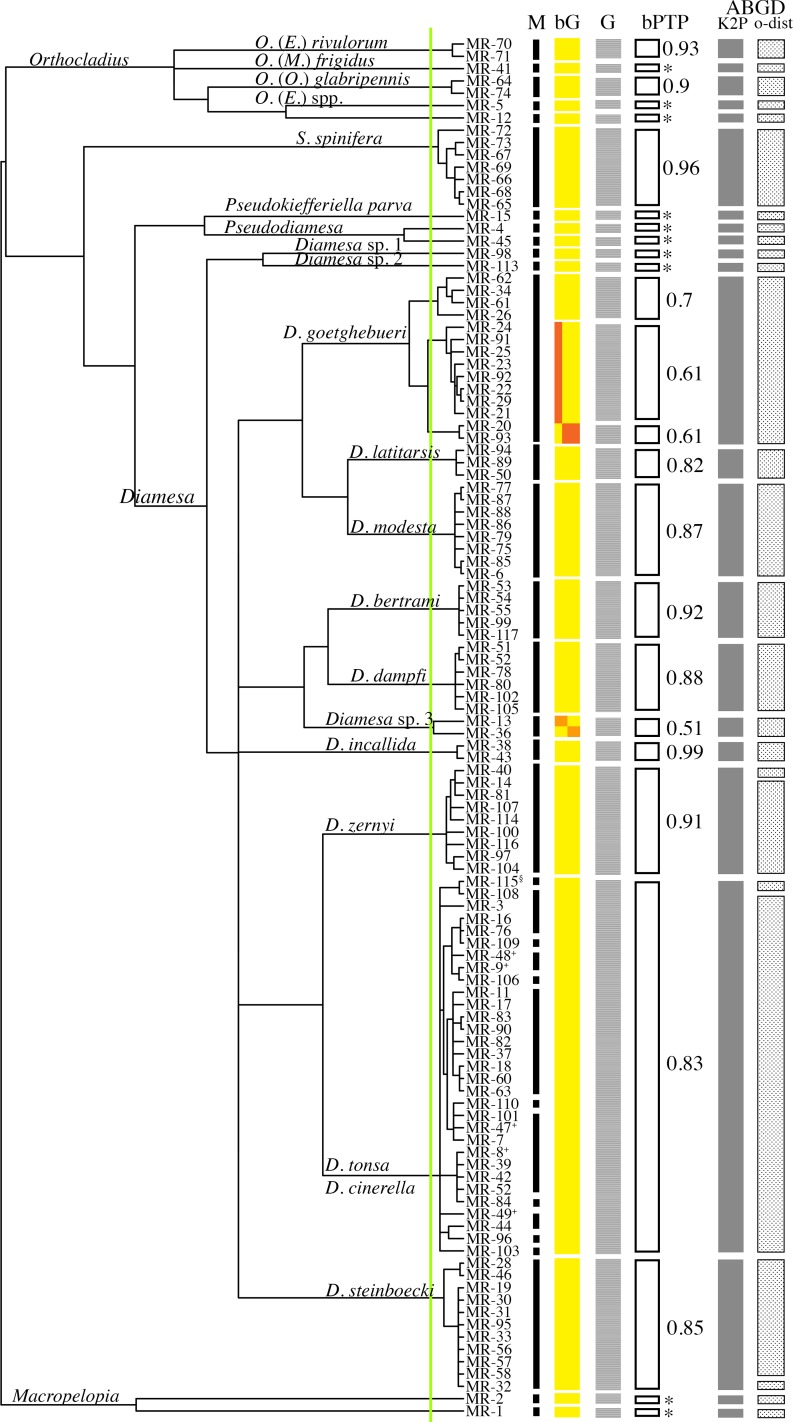
Species delimitation analysis based on *cox1* gene sequences. **A** Bayesian ultrametric tree inferred from the *cox1* gene sequence dataset and used as input for GMYC and bGMYC models. Specimen identifiers are reported on tips (MR as an acronym of the collection identifier plus the id number); ^§^: possible hybrid specimens between *D*. *vaillanti* and *D*. *tonsa*; ^+^: larvae at third instar. The vertical green line identifies the between/within species GMYC threshold. M: vertical black lines indicating the identified morphospecies. bG: putative species identified by bGMYC are represented by vertical solid colored boxes, colors indicate support values of Bayesian posterior probability (bpp) as follow: 0.05–0.5 in red, 0.5–0.9 in orange and 0.95–1 in yellow. G: vertical solid light-grey boxes represent putative species identified by GMYC. bPTP: black-edged boxes indicate the putative species (corresponding to the maximum likelihood partition) identified by the bPTP approach; values of bpp supporting putative species are reported, * = bpp of 1. Solid dark grey and light grey texture boxes indicate putative species identified by the ABGD approach, respectively implementing K2P and observed pairwise distance.

Species delimitation analyses performed by implementing the coalescent tree-based approach (i.e., GMYC, bGMYC and bPTP) led to almost identical results but some differences were apparent relative to ABGD (Fig 3). The GMYC model exhibited a significantly better likelihood than the null model (p-value < 0.001; logL_GMYC_ = 612.6, logL_NULL_ = 575.5), indicating that a boundary between and within species was identified. Twenty-five maximum likelihood entities (95% CI [24,26]) were identified at the threshold between Yule and Coalescent models (Fig 3). Similar results were obtained by bGMYC, which identified 25–26 evolutionary units, and by the bPTP method with 26 maximum likelihood partitions (estimated number of species between 23 and 34, average 26.2) (Fig 3).

**Fig 4:**
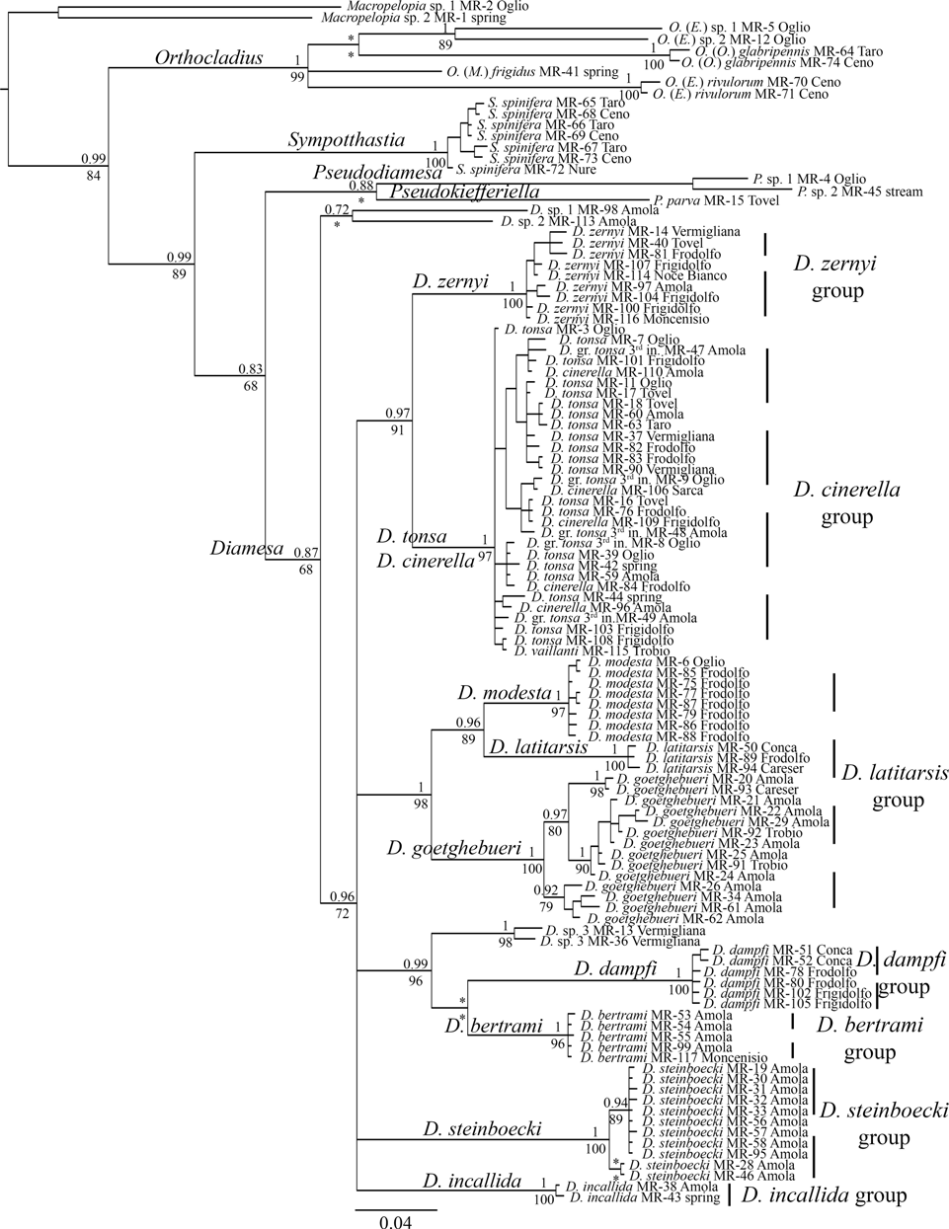
Bayesian consensus tree inferred from an alignment of 112 *cox1* gene sequences. On the nodes of main the lineages the support values are expressed as bpp (above) and aLRT (below); * denotes support values ≤ 0.65 bpp and ≤ 65% aLRT. Vertical dashed lines indicate species groups. The scale bar at the bottom indicates the distance in substitutions per site.

**Fig 5:**
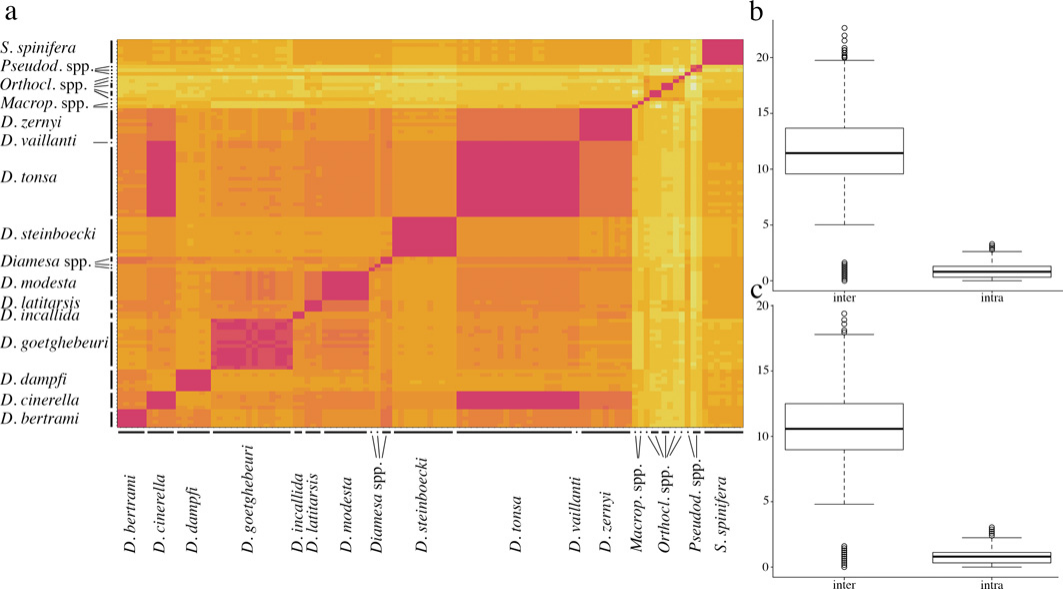
Pairwise Kimura two-parameter nucleotide distance. a. Heat map of the K2P pairwise distance matrix; values of nucleotide distance are proportional to color intensity, with low and high values of pairwise nucleotide distance indicated respectively by dark and light colors; morphological species are reported on the axis. Box-plot representing intra- and inter-specific K2P (b) and observed (c) pairwise nucleotide distance.

Discrepancy relative to the distance-based ABGD was recovered for specimens of *D*. *goetghebueri*, for which three separate evolutionary units were identified. No complete congruence between collecting localities and clustering pattern was recovered. Indeed, at the Amola glacier organisms belonging to all three identified evolutionary units of *D*. *goetghebueri* coexist in sympatry, whereas at Trobio and Careser only specimens belonging to one unit were found. The result that at Amola at least three independent lineages of *D*. *goetghebueri* coexist, which do not possess a recent common ancestor, could be interpreted as the result of the antiquity of this population or as the result of repeated events of colonization by organisms from different populations. Due to the small sample size and to the use of a single-gene marker, we cannot reach any reliable conclusions on the basis of the present data. A possible alternative scenario could be that larval stages of species phylogenetically close to *D*. *goetghebueri*, such as *D*. *lindrothi* Goetghebuer 1931 and *D*. *laticauda* Serra-Tosio 1964, are not distinguishable by currently-used morphological characters but segregate at the molecular level. At the lower value of the GMYC confidence interval (24 entities) two evolutionary units of *D*. *goetghebueri* collapse, while at the higher value (26 entities) the specimens MR-13 and MR-36 are recovered as separate units. Regarding the specimens belonging to *zernyi* group, GMYC and bGMYC achieved the same results obtained by ABGD, clearly discriminating specimens of *D*. *zernyi* (belonging to the same evolutionary unit) by the unit composed of *D*. *tonsa*, *D*. *cinerella* and *D*. *vaillanti* specimens. In addition, the same cases of discordance were confirmed: MR-14 and MR-40 identified initially as *D*. *tonsa*, and MR-115 a male pupa identified as *D*. *vaillanti*. The new morphological character (head capsule color gradient) developed in the present work (see paragraph below) revealed that MR-14 and MR-40 should be considered as *D*. *zernyi*. Morphological identification of specimen MR-115 is confirmed; nevertheless, as this organism exhibits contrasting characters it has been reported as a possible hybrid between *D*. *vaillanti* and *D*. *tonsa* (Fig 2; see paragraph below). Interestingly, on the basis of *cox1* sequences, it is not possible to discriminate between specimens of *D*. *tonsa* and *D*. *cinerella*: the two taxa were determined to be paraphyletic on the basis of the *cox1* gene (Figs 3 and 4) and possess values of pairwise K2P nucleotide distance (average 0.94%, SD = 0.22%) comparable with the average of intraspecific nucleotide distance (K2P-intra_avg_ = 0.88%, SD = 0.64%; K2P-inter_avg_ = 11.79%, SD = 3.58%; Fig 5, Table 2). The graphical representation of pairwise K2P nucleotide distance matrix through the heat map allows the identification of a group of specimens on the basis of their pairwise K2P nucleotide distance values (Fig 5A). Comparisons between morphologically conspecific specimens are denoted by darker boxes (low values of pairwise nucleotide distance) while non-conspecific comparisons, with some already discussed exceptions, are characterized by light boxes (high values of pairwise nucleotide distance; Fig 5). The non-overlapping distribution of intra- and inter-specific pairwise K2P/observed distances confirmed the existence of a clear gap in pairwise comparisons (box plots in Fig 5A–5C).

**Table 2:** Within and between Kimura 2 parameter nucleotides mean distances and mean values of observed nucleotide differences^a^.

*Diamesa* species	*cinerella*	*tonsa*	*zernyi*	*modesta*	*latitarsis*	*goetghebueri*	*dampfi*	*bertrami*	*steinboecki*	*incallida*	*vaillanti*
*cinerella* (5)	**0.9(0.2)**	6.2	39.9	51.1	53	57	68.8	54.5	59.4	57.1	6.4
*tonsa* (20)	0.9(0.2)	**0.9(0.2)**	40.2	51.8	53.5	58	68.7	54.2	59.8	58.1	6.4
*zernyi* (9)	6.3(0.9)	6.4(0.9)	**0.8(0.2)**	61.4	57.7	64	68.3	55.3	66.4	56.8	38.4
*modesta* (8)	8.2(1.1)	8.4(1.1)	10(1.2)	**0.2(0.1)**	41.6	47.3	68.6	64.4	73.8	59.6	48.5
*latitarsis* (3)	8.5(1.0)	8.6(1.1)	9.3(1.1)	6.6(1)	**0.4(0.2)**	56.6	68.3	55.2	68.3	57.5	50
*goetghebueri* (14)	9.2(1.2)	9.4(1.2)	10.4(1.2)	7.6(1)	9.2(1.1)	**1.7(0.3)**	69.5	66.7	69.7	61.4	55.4
*dampfi* (6)	11.3(1.3)	11.3(1.3)	11.2(1.3)	11.3(1.4)	11.2(1.4)	11.5(1.4)	**0.4(0.2)**	56.9	75.3	69.2	68.2
*bertrami* (5)	8.8(1.1)	8.8(1.1)	8.9(1.1)	10.5(1.2)	8.9(1.1)	10.9(1.3)	9.2(1.2)	**0.1(0.1)**	68	64.3	54
*steinboecki* (11)	9.7(1.2)	9.8(1.2)	10.9(1.3)	12.2(1.4)	11.2(1.3)	11.5(1.3)	12.6(1.4)	11.2(1.2)	**0.4(0.1)**	73.3	59.5
*incallida* (2)	9.2(1.1)	9.32(1.15)	9.2(1.2)	9.7(1.3)	9.3(1.2)	10(1.3)	11.4(1.4)	10.5(1.3)	12.1(1.4)	**0.2(0.1)**	57.5
*vaillanti* (1)	1(0.3)	1(0.3)	6.1(0.9)	7.8(1.1)	8(1)	8.9(1.2)	11.2(1.3)	8.7(1.1)	9.7(1.2)	9.3(1.2)	**-**

^a^ Distances are expressed as percentages.

Below the diagonal are reported mean values of K2P distance between-taxa calculated on *cox1* gene; on the diagonal, mean values of within-taxa K2P distance are reported in bold. Above the diagonal are reported the mean values of the observed nucleotide differences between taxa. Standard deviations are reported within parentheses.

### Topology Inferred from Cox1 Gene Sequences

Although a single DNA marker can fail to produce a reliable phylogeny among organisms, we believe that the results achieved in the present study on the basis of *cox1* gene sequences have merit. A Bayesian consensus tree was inferred as an input for the tree-based species delimitation methods (GMYC, bGMYC and bPTP; Fig 4). Interestingly, the inferred topology clearly determines the close relationships of taxa belonging to the same species group, as defined by morphological synapomorphies exhibited by males and pupal exuviae [13]. All the determined species groups were well supported (BI≥0.97, aLRT≥91). In contrast, relationships among the species groups are not resolved by *cox1* gene sequences. These results could be explained by the limitation of *cox1* in recovering the cladogenesis among the species groups under analysis or, alternatively, that almost simultaneous cladogenetic events led to the formation of the main species groups of *Diamesa*. The approach adopted in the present study is not adequate to test the latter hypothesis; further investigations is currently in progress.

On the basis of *cox1* gene sequences over a total of 16 analyzed morphospecies, 11 were monophyletic while three are represented by a single specimen; the two sympatric species *D*. *tonsa–D*. *cinerella* were paraphyletic and clustered in a single group. The branching pattern obtained for *D*. *tonsa–D*. *cinerella* is congruent with a scenario of recent origin for these two taxa, in which a complete lineage sorting has not yet been achieved; episodes of gene flow between these species represent a further possible explanation. The clear differences in the morphology of male genitalia, associated with a small between-taxa nucleotide mean distance (0.94%, SD = 0.22%; Fig 5A, Table 2), is further evidence that morphological features could evolve more rapidly than neutral/semi neutral genetic markers [57–60].

### New Diagnostic Character and Image Analysis

**Fig 6:**
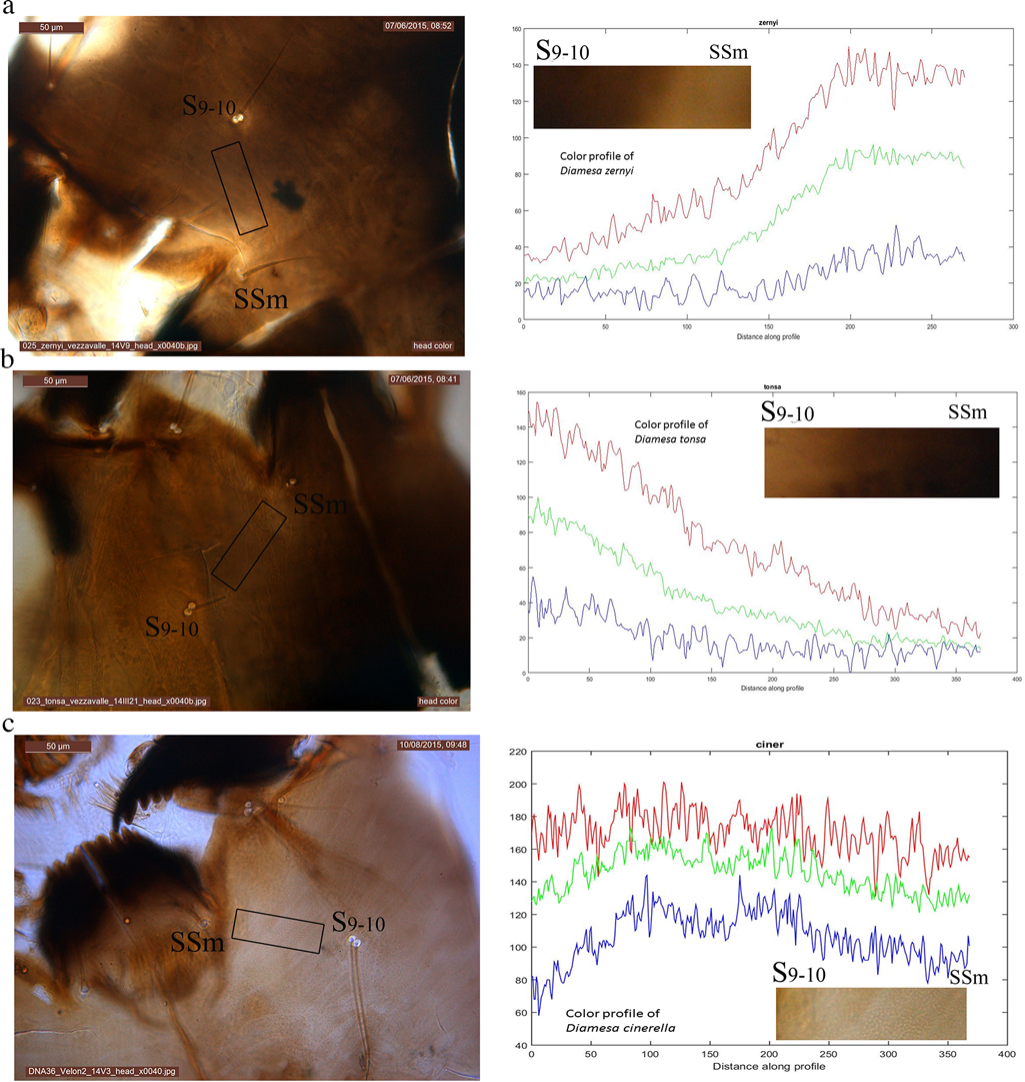
A novel morphological diagnostic character: color gradient between submenti and genal setae. Photo of *Diamesa* head capsule, the area of interest is highlighted by a rectangle. For each species on the left side is reported a micrograph of the head capsule; on the right side a graph reporting the RGB color profile of the analyzed region, embedded in the graph a picture reporting the color gradient from the analyzed specimens. A. *Diamesa zernyi*. B. *Diamesa tonsa*. C. *Diamesa cinerella*. SSm: setae submenti; S_9-10_: genal setae.

The cases of discrepancies observed within *zernyi* and *cinerella* groups with regard to morphological and molecular identification methods prompted us to re-analyse slides of all specimens to accurately explore the color of the head capsule. Detailed analyses lead to the discovery of a new and, in our view, more accurate diagnostic character represented by the color gradient from the genal setae to the setae submenti (Fig 6). Reared larvae of *D*. *zernyi* exhibit a color profile from a darker color in the genal region (from genal setae) to a lighter color in the submental region (from submenti setae) (Fig 6A), whereas those of *D*. *tonsa* exhibit the opposite trend with a lighter color in the genal region and a darker color in the submental region (Fig 6B). The color gradient was not observed in reared larvae of *D*. *cinerella*: indeed they possess a consistent pale color in both genal and submental regions (Fig 6C).

All specimens belonging to the *zernyi* group were then re-analyzed and identified according to the newly developed character. Interestingly, all specimens previously identified as not clustering with conspecific specimens were misidentified according with the newly discovered character. Only the identification of mature male pupa MR-115, assigned on the basis of hypopygium to *D*. *vaillanti*, but clustering within the *D*. *tonsa*–*D*. *cinerella* clade on the basis of the *cox1* gene sequence, produced a conflict. On the basis of the achieved results and considering the sister relationship between *D*. *zernyi* and *D*. *vaillanti* [61] we can hypothesize that the specimen MR-115 represents a hybrid between *D*. *tonsa/D*. *cinerella* and *D*. *vaillanti*. This result does not affect the status of the species since the capability of closely related taxa to hybridize is regarded as a plesiomorphic state that occurs among insects (e.g. [62, 63]) and it has been demonstrated in Chironomidae (e.g., [64, 65]). Crucially, the possible event of hybridization occurred at a very small glacier (area < 1 km^2^), the Trobio, in the Orobian Alps, where the limited living and breeding habitats improve the probability of contact amongst organisms. Analyses that include information provided by nuclear genes are required to rigorously test the possible hybridization event.

### DNA Barcoding

A total of six sequence datasets were analyzed. Features and DNA barcoding performances of each dataset are reported in Table 3. The analyses for the estimation of intra-/inter- specific nucleotide OT achieved contrasting results depending on the dataset analyzed (Table 3). The estimated OT ranges from a minimum value of 0.7% in the case of *ds2*, where sequences of larvae at the 3^rd^ developmental stage and singletons were excluded, to a maximum value of 1.4% for *ds1*, where only sequences of larvae at the 3^rd^ developmental stage were excluded. Values of OT estimated from Alpine non-biting midges included in this study were much lower than those obtained for the delineation of species belonging to the genus *Tanytarsus* (4–5%; [18]). For the estimated OTs the cumulative error of misidentification ranges from 0, in the case of *ds4* and *ds6*, to 26 in the case of *ds1*. Twenty-five out of the 26 misidentifications are due to specimens morphologically identified as *D*. *tonsa* and *D*. *cinerella*. Previous results can be explained by: *i*) the value of pairwise K2P nucleotide mean distance between the two species (0.94%; Table 2, Fig 5) being lower than the estimated OT (1.4% in the case of *ds1*), and by *ii*) the paraphyletic status of the two species on the base of *cox1* gene sequences (Figs 3 and 4). The remaining case of misidentification is represented by the apparent hybrid between *D*. *vaillanti* and *D*. *tonsa*. Nevertheless, the near neighbor analysis [66] highlighted that the majority of tested sequences (from ~80% to 100%) showed a conspecific individual as closest. Overall, the DNA barcoding approach on the six analyzed datasets achieved on average an accuracy of 89% [74%, 100%] and a precision of 99% [92%, 100%]. Results obtained by the DNA barcoding approach on the midge species under study here are very promising and confirm its enormous utility in supporting rapid and large-scale for the evaluation of insect biodiversity of high-altitude stream habitats.

**Table 3:** DNA Barcoding statistics and performances.

ID^a^	Sets^b^	Excluded^c^	N^d^	eOT^e^	CEeOT^f^	NN^g^	A^h^	P^i^
*ds1* (107)		L3	24 (8) [1,20]	1.4–4.8	26 (0, 26)	86T, 21F	76	100
*ds2* (95)	ds2 ⊂ ds1	L3, sng	13, 7 [2,20]	0.7–0.8	25 (6,19/0,25)	84T, 11F	74	92
*ds3* (90)	ds3 ⊂ ds2	L3, sng, *D*. *cinerella*	12, 7 [2,20]	0.8,1.0	9 (0,9/1,8)	88T, 2F	90	99/100
*ds4* (89)	ds4 ⊂ ds3	L3, sng, *D*. *cinerella*, hybrid	12, 7 [2,20]	1.0–5.3	0 (0,0)	89T	100	100
*ds5* (75)	ds5 ⊂ ds2	L3, sng, *D*.*tonsa*	12, 6 [2,14]	0.8	3 (0,3)	73T, 2F	96	100
*ds6* (74)	ds6 ⊂ ds5	L3, sng, *D*. *tonsa*, hybrid	12, 6 [2,14]	0.8–5.2	0 (0,0)	74T	100	100

^a^ Identifier of each analyzed datasets.

^b^ Logical relation among datasets.

^c^ List of excluded *cox1* sequences respect to the 112 obtained; L3: larvae at 3^rd^ developmental stage; sng: singletons; hybrid: hybrid specimen between *D*. *vaillanti* and *D*. *tonsa*.

^d^ Number of morphospecies included in the dataset; within brackets the average number of specimens per species; within square brackets the minimum and maximum number of specimens per species.

^e^ Estimated optimal threshold: nucleotide distance or range of distances, expressed as percentage, that minimize the function *F*_*x*_ = min∑ (FP+ FN).

^f^ CEeOT: cumulative error at eOT, within brackets are reported the number of FP and FN.

^g^ Near neighbor defined as Maier et al. (2006), number of tested sequences with as closest individual a conspecific (true, T) or a non conspecific specimens (false, F).

^h^ Accuracy calculated as follow: A = (TP+TN)/n° sequences; values are expressed as percentage.

^i^ Precision calculated as follow: P = TP/(TP+FP); values are expressed as percentage. FP: false positive identification corresponding to type I errors; FN: false negative corresponding to type II errors; TP: true positive; TN: true negative.

## Conclusion

The present study, mainly focused on testing the congruence between species identified using “traditional” morphological characters and putative molecular species (identified by a ~650 bp fragment of the mitochondrial cytochrome oxidase I), has demonstrated an almost complete congruence in the results achieved by both approaches. Cases of discrepancies between the two methods were recovered within *zernyi* and *cinerella* groups, where some larval specimens were found to be misidentified on the basis of the traditionally used morphological characters (i.e., the overall color of the head capsule) and a possible hybrid between *D*. *vaillanti* and *D*. *tonsa* was collected at Trobio glacier. In addition, identification methods based on *cox1* gene sequences failed to distinguish between specimens belonging to the clade *D*. *tonsa*–*D*. *cinerella* as these two taxa were found to be paraphyletic on the base of this marker. Further analyses, based on a multi-gene approach or on more innovative methods such as RAD sequencing, are required to disentangle the intricate relationships between these two sister species.

The above-mentioned critical cases determined within *zernyi* and *cinerella* groups prompted us to analyze more carefully the color of the head capsule. This larval character, despite being influenced by several factors such as the developmental stage and specimens conservation and preparation, has been extensively used to identify larvae of *Diamesa* [12, 15, 16]. The procedure led to the discovery of a more accurate trait related to head capsule color. Larvae of *D*. *zernyi*, *D*. *tonsa* and *D*. *cinerella*, in the 4^th^ developmental stage, are more accurately identifiable on the base of the color gradient between the setae submenti and genal setae. This character, focusing on the color gradient, in influenced to a lesser extent, with respect to overall head color, by storage conditions and specimens preparation. We believe that it is important to remark that the discovery of the new diagnostic character has been possible only because species delimitation analyses performed on molecular data highlighted cases in which information provided by molecules were in contrast with those supported by morphology. This result represents further evidence that “traditional” taxonomy benefits from the molecular tools and that conclusive results can only be achieved by adopting integrated approaches.

On average, performances of molecular identifications through DNA barcoding were found to be elevated, with a recovered accuracy in specimen identification of ~89% and a precision of ~99%. These values reached 100% after the removal of specimens identified as *D*. *tonsa* or as *D*. *cinerella* and of the possible hybrid specimen recovered at the Trobio glacier. The results allow us to conclude that the *cox1* gene sequence is an useful aid in species identification and paves the way for the use of molecular taxonomy, through DNA barcoding or DNA metabarcoding protocols, in support of biological studies aiming to monitor and evaluate the biodiversity of midges, and more generally invertebrates, inhabiting high-altitude streams and cold spring habitats. Nevertheless, it is fair to remember that in some cases (i.e., *D*. *tonsa* and *D*. *cinerella*) molecular tools fail in specimen identification, and thus the support of specialist entomologists is still required.

## Supporting Information

S1 File Automatic Barcode Gap Discovery analysis. Histogram of pairwise nucleotide distances (Fig A). Plot of ranked pairwise nucleotide distances (Fig B). Automatic partition of the analyzed data set (Fig C).
(DOCX)
