# Correction: Integrated Taxonomy and DNA Barcoding of Alpine Midges (Diptera: Chironomidae)

**DOI:** 10.1371/journal.pone.0159124

**Published:** 2016-07-07

**Authors:** Matteo Montagna, Valeria Mereghetti, Valeria Lencioni, Bruno Rossaro

The following information is missing from the Data Availability statement: Sequence data are deposited at ENA archive (accession numbers: LN897576- LN897687). Morphological voucher specimens (MR-1 to MR-117) are deposited in Bruno Rossaro collection at Dipartimento di Scienze degli Alimenti—Università degli Studi di Milano and in the MUSE-Museo delle Scienze entomological collections (Valeria Lencioni collection).
