# Supplementary material for: Integrated Taxonomy and DNA Barcoding of Alpine Midges (Diptera: Chironomidae)
Source: PLoS One. 2016 Mar 3;11(3):e0149673. doi: 10.1371/journal.pone.0149673 (PMC4777558; doi:10.1371/journal.pone.0149673)

**SUPPLEMENTARY FIGURES for the manuscript**

**Integrated taxonomy and DNA barcoding of Alpine midges (Diptera: Chironomidae)**

**Matteo Montagna, Valeria Mereghetti, Valeria Lencioni & Bruno Rossaro**

Submitted to PLOS ONE as a Research Article

This file includes:

Supplementary Figures A, B, C

**Figure A.** Histogram of pairwise nucleotide distances. Graph obtained through the web-based interface available at (http://wwwabi.snv.jussieu.fr/public/abgd); model of nucleotide evolution settled to Kimura-2-parameter [40].


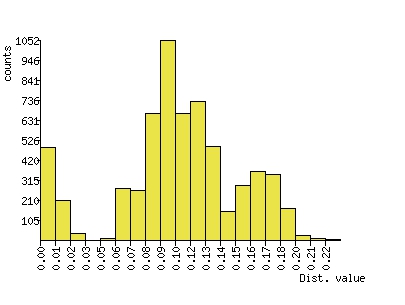


**Figure B.** Plot of ranked pairwise nucleotide distances. Graph obtained through the web-based interface available at (http://wwwabi.snv.jussieu.fr/public/abgd); model of nucleotide evolution settled to Kimura-2-parameter [40].


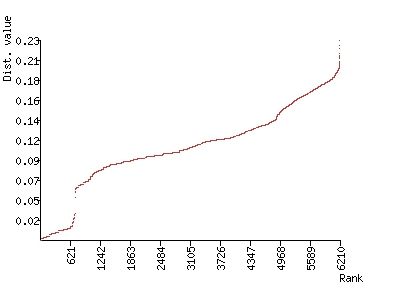


**Figure C.** Automatic partition of the analyzed data set. Graph obtained through the web-based interface available at (http://wwwabi.snv.jussieu.fr/public/abgd). The number of groups composing the partitions (initial and recursive) are reported as a function of the prior limit between intra- and interspecies divergence.


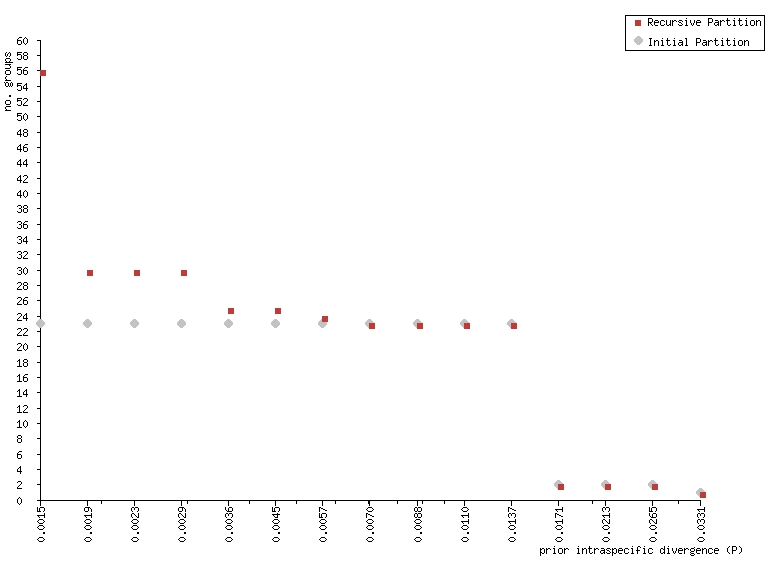

Supplement: S1 File — Histogram of pairwise nucleotide distances (Fig A). Plot of ranked pairwise nucleotide distances (Fig B). Automatic partition of the analyzed data set (Fig C). (DOCX) [file pone.0149673.s001.docx]
